# Screening and triage of intrauterine growth restriction (IUGR) in general population and high risk pregnancies: a systematic review with a focus on reduction of IUGR related stillbirths

**DOI:** 10.1186/1471-2458-11-S3-S1

**Published:** 2011-04-13

**Authors:** Aamer Imdad, Mohammad Yawar Yakoob, Saad Siddiqui, Zulfiqar Ahmed Bhutta

**Affiliations:** 1 Division of Women and Child Health, The Aga Khan University, Stadium Road, P.O. Box 3500, Karachi-74800, Pakistan

## Abstract

**Background:**

There is a strong association between stillbirth and fetal growth restriction. Early detection and management of IUGR can lead to reduce related morbidity and mortality. In this paper we have reviewed effectiveness of fetal movement monitoring and Doppler velocimetry for the detection and surveillance of high risk pregnancies and the effect of this on prevention of stillbirths. We have also reviewed effect of maternal body mass index (BMI) screening, symphysial-fundal height measurement and targeted ultrasound in detection and triage of IUGR in the community.

**Methods:**

We systematically reviewed all published literature to identify studies related to our interventions. We searched PubMed, Cochrane Library, and all World Health Organization Regional Databases and included publications in any language. Quality of available evidence was assessed using GRADE criteria. Recommendations were made for the Lives Saved Tool (LiST) based on rules developed by the Child Health Epidemiology Group. Given the paucity of evidence related to the effect of detection and management of IUGR on stillbirths, we undertook Delphi based evaluation from experts in the field.

**Results:**

There was insufficient evidence to recommend against or in favor of routine use of fetal movement monitoring for fetal well being. (1) Detection and triage of IUGR with the help of (1a) maternal BMI screening, (1b) symphysial-fundal height measurement and (1c) targeted ultrasound can be an effective method of reducing IUGR related perinatal morbidity and mortality. Pooled results from sixteen studies shows that Doppler velocimetry of umbilical and fetal arteries in ‘high risk’ pregnancies, coupled with the appropriate intervention, can reduce perinatal mortality by 29 % [RR 0.71, 95 % CI 0.52-0.98]. Pooled results for impact on stillbirth showed a reduction of 35 % [RR 0.65, 95 % CI 0.41-1.04]; however, the results did not reach the conventional limits of statistical significance. This intervention could be potentially recommended for high income settings or middle income countries with improving rates and standards of facility based care. Based on the Delphi, a combination of screening with maternal BMI, Symphysis fundal height and targeted ultrasound followed by the appropriate management could potentially reduce antepartum and intrapartum stillbirth by 20% respectively. This estimate is presently being recommended for inclusion in the LiST.

**Conclusion:**

There is insufficient evidence to recommend in favor or against fetal movement counting for routine use for testing fetal well being. Doppler velocimetry of umbilical and fetal arteries and appropriate intervention is associated with 29 % (95 % CI 2% to 48 %) reduction in perinatal mortality. Expert opinion suggests that detection and management of IUGR with the help of maternal BMI, symphysial-fundal height measurement and targeted ultrasound could be effective in reducing IUGR related stillbirths by 20%.

## Background

Intrauterine growth restriction (IUGR) represents pathological inhibition of fetal growth and failure of the fetus to attain its growth potential [1]. There is a strong association between stillbirth and fetal growth restriction [2]. The etiology and risk factors for stillbirth and IUGR largely overlap [3,4]. Both the conditions are the result of complex pathology resulting from a recognizable interaction among maternal conditions, placental dysfunction and hormonal regulation [2,4]. For example, maternal smoking, low educational level, advanced maternal age, nulliparity, and black race are associated with increased risk of fetal growth restriction and stillbirth [2,4,5]. The same is the case for maternal medical conditions like gestational hypertensive disorders, pre and gestational diabetes, systemic lupus erythematosus, chronic renal disease, and thyroid disorders [2,3,6]. Further evidence of strong association between IUGR and stillbirth comes from the fact that prior delivery of a growth restricted infant is among the strongest risk factors for stillbirth, comparable to the history of prior stillbirth[3].

IUGR has been used as a marker to assess complications of pregnancy [7]. There is however, no standard definition of IUGR. It has been defined as a birth weight < 2 standard deviations below the median for gestational age, whereas others use a threshold of 3rd or 5th percentile of weight for age for the given population [7,8]. The term small for gestational age (SGA), usually defined as having a birth weight below the 10th percentile of an accepted reference standard, is often used as a proxy measure for IUGR [8]. These two terms are however not synonymous as some SGA infants may merely represent the lower tail of the 'normal' fetal growth distribution, while others who have been affected *in utero* by an inadequate nutritional milieu or other growth-inhibiting influences may nevertheless have a birth weight that is 'appropriate' for gestational age (AGA) [8]. Even though the terms SGA and IUGR are not synonymous, there is correlation between the two and the higher the SGA rate, the greater the likelihood that SGA is a result of IUGR [9].

According to an estimate, approximately 30 million newborns per year are affected with intrauterine growth restriction in developing countries [4]. This rate is six times higher than that in developed countries. The highest burden of prevalence of SGA/IUGR babies lies in Asia (75%), mainly South East Asia, followed by Africa (20%) and Latin America (5%) [4].

In order to prevent complications associated with intrauterine growth restriction, it is important to first detect the condition and once detected, institute appropriate surveillance to asses fetal well being coupled with suitable intervention in case of fetal distress (for example early delivery) [1,10]. The primary purpose of this paper is to assess screening and surveillance interventions that can help prevent stillbirths associated with IUGR. In this paper we review the methods used to detect IUGR followed by the methods used for surveillance of such high risk pregnancies. This paper is part of series of papers to estimate effectiveness of an intervention for input to Lives Saved Tool (LiST) model [11]. An intervention is currently included in the LiST if there is evidence that it reduces maternal mortality, infant/child mortality (<5 years) and/or stillbirths. The process of generating recommendations for an intervention involve qualitative evaluation of available evidence according to GRADE criteria [12] and quantitative evaluation according to Child Health Epidemiology Reference Group (CHERG) rules [11]. For more details of the review methods, the adapted GRADE approach or the LiST model see the methods section and the CHERG method paper [11]. For the purpose of simplicity, we will divide this review into parts.

1. The detection of IUGR.

2. Surveillance of high risk pregnancies.

## Methods

### Search strategy

We systematically reviewed all published literature to identify studies evaluating role of (1) fetal movement monitoring and (2) Doppler ultrasound in high risk pregnancies in reducing perinatal mortality and stillbirths. We searched PubMed, Cochrane Library, and all World Health Organization Regional Databases and included publications in any language. The search strategies used for the above mentioned screening strategies are given in Additional file 1. Last date of search was 3^rd^ March 2010. We scanned the titles and abstracts of the studies identified to exclude those that were obviously irrelevant, retrieved the full text of the remaining studies, and identified relevant articles. We also reviewed the reference lists of identified articles, existing reviews and meta-analyses and looked for studies that were not picked up in the main search. Authors were contacted for any additional data, if required.

#### Inclusion/exclusion criteria

For Doppler velocimetry, only randomized trials and quasi-randomized studies addressing the use of Doppler ultrasound in ‘high risk’ pregnancies were considered for inclusion in the review. Women with ‘high risk’ pregnancies were defined as those women with singleton or twin pregnancy in which the maternal or fetal condition could be expected to lead to fetal compromise, e.g. identified intrauterine growth restriction, post-term pregnancies, previous pregnancy loss, women with hypertension, diabetes or other maternal pathology (e.g. thrombophilia) [13]. Only those studies have been considered for inclusion in the review in which Doppler ultrasound of fetal and umbilical vessels was performed. Studies addressing utero-placental circulation were excluded however where umbilical artery or fetal Doppler was combined with utero-placental Doppler, the study has been included in this review.

For the fetal movement monitoring, we included randomized controlled trials, quasi-randomized and observational studies. The included studies either compared different methods of fetal movement monitoring vs. no fetal movement monitoring, mixed or undefined monitoring. Studies addressing effectiveness of fetal movement counting in high risk pregnancies and/or unselected populations were considered.

#### Data abstraction and validity assessment

All relevant data from final studies were abstracted on a standardized Excel spreadsheet (Additional file 2). Key variables extracted included study design, setting, allocation concealment, blinding, loss to follow-up, details of the intervention and comparison groups and the outcomes. The studies were assessed and graded according to the CHERG adaptation of the GRADE technique [12]. This method of assessment is based on strengths and limitations of individual studies. The studies are graded as ‘high’ ‘moderate’ ‘low’ or ‘very low’ quality based on study design, study quality, relevance to the objectives of the review and consistency across studies [11]. A randomized or cluster randomized trial initially received a high score which was downgraded to moderate if study design limitations or biases were present. In addition, studies having intent-to-treat analysis or a statistically significant strong association received 1-2 grade increases. Any study with a final grade of ‘very low’ was excluded from the analysis.

### Quantitative data synthesis

We generated meta-analyses where data were available from more than one study and intervention and control groups did not have gross clinical heterogeneity. The primary outcome was stillbirths and/or perinatal death. The main comparison for Doppler velocimetry studies was Doppler ultrasound of fetal vessels versus no Doppler ultrasound of fetal vessels (including comparisons of Doppler ultrasound of fetal vessels concealed versus Doppler ultrasound of fetal vessels revealed). For cluster randomized trials, we used the stated cluster adjusted relative risk and 95% confidence interval, irrespective of the method used. We adjusted the results for cluster design if not stated in the study. The assessment of statistical heterogeneity among trials was done by visual inspection i.e. the overlap of the confidence intervals among the studies, and by the Chi square (P-value) of heterogeneity in the meta-analyses and I^2^ value. A low P value (less than 0.10) or a large chi-squared statistic relative to its degree of freedom (I^2^ >50 %) was considered as providing evidence of significant heterogeneity. In situations of substantial or high heterogeneity being present, causes were explored by sensitivity analysis. Fixed models were used for the primary analysis. All meta-analyses were conducted using software Review Manager Version 5 [14].

For recommendations to the LiST model, we summarized the evidence for each outcome including qualitative assessment of ‘overall’ evidence according to GRADE criteria and quantitative measures according to standard guidelines of Child Health Epidemiological Review Group (CHERG) group [11]. The qualitative evaluation of the overall (pooled) evidence was based on the volume and consistency of the evidence across studies, the size of pooled relative risk and the strength of the statistical evidence for an association between the intervention and the health outcome as reflected in the p-value [11].

### Delphi process for establishing expert consensus

We did the Delphi process for generation of effect estimates for detection of IUGR by a proposed package that includes i) maternal BMI screening, ii) symphysis-fundal height measurement and iii) targeted ultrasound. This process involves consultation with experts in the field and asks their opinion about the effectiveness of an intervention [11]. The panel invited to participate were experts in newborn health and sepsis representing six WHO regions (South Asia, Africa, Western Europe, Eastern Europe, North America, Australia), and including multiple disciplines international health, obstetrics/gynecology/midwifery etc. Thirty-one experts agreed to participate in the Delphi process. The questionnaire was developed by MYY and ZAB, and refined after several rounds of pilot testing. The questionnaire was sent by email and included the background and aims of the Delphi and estimates of effect that were available from the literature for different scenarios. The median response and range were determined for each question. Consensus was defined a priori as an interquartile range in responses of < 30% for each question. For those estimates not reaching consensus, the plan was for results to be electronically distributed to the panel, virtual discussion allowed, and a second round of email questionnaires sent. However, consensus was achieved after one round of questionnaires and subsequent rounds were not considered necessary.

## Results

### The detection of IUGR

This section will summarize the previous evidence and selection of interventions for detection of IUGR in the general population.

Some of the methods used to predict and monitor growth of the fetus include maternal BMI screening, symphysis-fundal height measurement and routine ultrasound [15]. Maternal BMI screening had been proposed as an effective method of predicting fetal growth by a group of experts [16]. Two Cochrane reviews on routine ultrasonographic evaluation in early (before 24 weeks of gestation) and late pregnancy (after 24 weeks) showed no effect in reducing overall peri-natal mortality [17,18]. Early pregnancy ultrasound (before 24 weeks) however was beneficial in detecting multiple pregnancies and reducing rates of induction of labor for post-term pregnancies [18]. Another Cochrane review on effectiveness of symphysis-fundal height measurement was inconclusive as only one trial was included and no recommendations in favor or against of the intervention were made [19].

For detection of IUGR, our approach was based on the results of a previous review conducted by us on different screening interventions during pregnancy [15]. On the basis of this review and other related evidence, a set of three interventions was proposed [15,16]. These interventions include (a) maternal BMI screening, (b) symphysis-fundal height measurement and (c) targeted ultrasound. The current evidence for these interventions is described based on our previous review and a summary of results is presented below.

Maternal anthropometry can be used to help predict adverse perinatal outcomes including low birth weight and preterm birth [16,20]. Appropriate detection and management of maternal malnutrition can significantly reduce the occurrence of IUGR and related perinatal adverse outcomes [21]. One of the nutritional interventions that have a proven effect in reducing incidence of SGA/IUGR is balanced protein energy supplementation [5]. A Cochrane review by Kramer et al. on protein energy supplementation during pregnancy had shown that balanced protein energy supplementation can reduce occurrence of small for gestational age births by 32% [RR 0.68 (95 % CI 0.56, 0.84)] [22].

A Cochrane review by Nielson on effectiveness of symphysis-fundal height measurement was inconclusive as there was only trial that included 1369 women [19]. None of the outcomes measured was statistically significant. Even though there was no conclusive evidence from the only randomized trial, some observational studies report that symphysis-fundal height measurement can be a cost effective and relatively accurate method for measurement of gestational age and subsequently fetal growth. A recent cohort study conducted in Pakistan compared fundal height measurement with recall of last menstrual period (LMP) to assess gestational age [23]. The effectiveness of both the interventions was compared with ultrasound. The results showed that symphysis-fundal height measurement was a better method of assessing gestational age compared to recall of LMP, however accuracy of both the methods was less than that of ultrasound. Authors suggested use of symphysis-fundal height measurement as a cost effective and relatively reliable method of gestational age assessment and fetal growth monitoring. Another study reported that weekly self-administered symphysis-fundal measurements can be used to monitor fetal growth [24]. Similar results were found in an observation study from Brazil [25] where 753 low risk women were followed with periodic symphysis-fundal height measurement and the results were plotted to obtain a curve. Results showed a sensitivity of about 86 % for detection of SGA infants.

**Figure 1:**
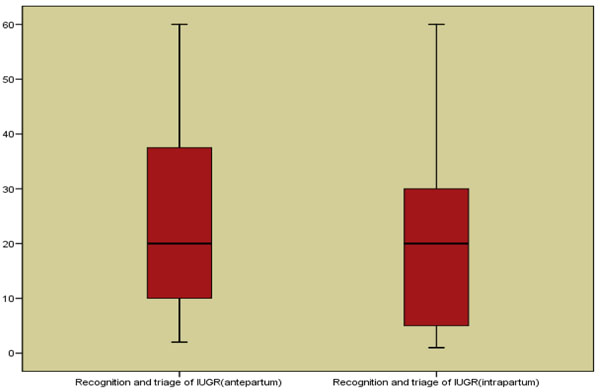
Box plots of the Delphi results on detection and management of IUGR compared to no identification or action for IUGR

Even though routine ultrasound early (< 24 weeks of gestation) or late (> 24 weeks) in pregnancy have not been shown to decrease perinatal mortality [17,18], repeated ultrasound estimation of growth can be used to detect abnormal fetal growth [10]. We propose that if this is combined with monitoring of fetal growth by symphysis-fundal height measurement and coupled with appropriate management (e.g. early delivery), it can substantially reduce perinatal mortality and stillbirth. We did a Delphi process to get an estimate for effectiveness of detection and management of IUGR for inclusion in the LiST [11]. In this process we contacted experts in the field and took their opinion on the effectiveness of IUGR screening using the above mentioned three methods (a-c) coupled with the appropriate management. The process revealed an estimated reduction of 20% each in antepartum and intrapartum stillbirths (Figure 1). This estimate had been recommended for inclusion in the LiST.

### Surveillance of high risk pregnancies

#### (1) Fetal movement monitoring

**Figure 2:**
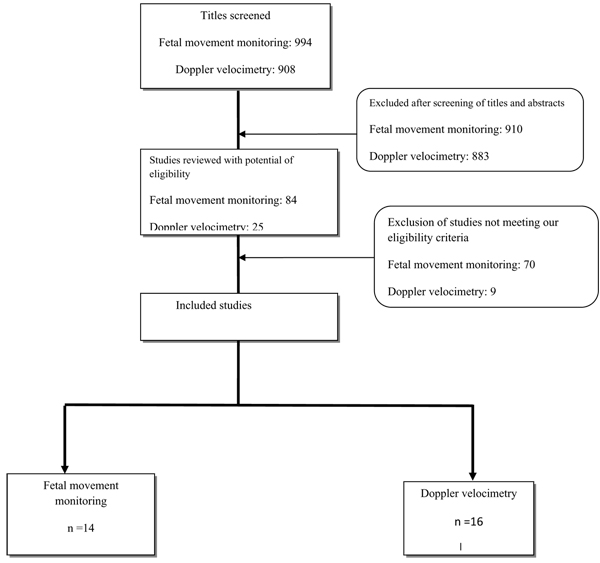
Synthesis of study identification in review of screening and triage of intrauterine growth restriction in general population and high risk pregnancies

During the literature search, a total of 994 titles were identified (Figure 2). After an initial screening of titles and abstracts, 84 were found to be appropriate and finally 14 studies were chosen for final data extraction. We evaluated studies on the basis of antepartum or intrapartum stillbirth and perinatal mortality as outcomes. Additional file 3 gives characteristics of included studies of fetal movement monitoring. There were four randomized controlled trials assessing fetal movement counting [26-29]. Three of these trials were conducted in developed countries [26,28,29] and one in a developing country [30]. Data were not pooled due to gross clinical heterogeneity in the assessment of fetal movement monitoring and the comparison group. Two of these trials compared different fetal movement counting methods, and measured the acceptability, the compliance and other outcomes [29,30]. No intrauterine death was reported in any of these two trials. In another trial fetal movement counting (modified Cardiff method) was compared with hormonal analysis. Only one stillbirth was reported (in the fetal counting group). However the fetal movement counting group had significantly fewer visits to the hospital antenatally compared to the group undergoing hormone analysis (RR 0.26, 95% CI 0.20 to 0.35). The fourth and largest trial, was a cluster randomized study by Grant et al. [26] involving 68,654 women comparing fetal counting (Cardiff method) versus no instruction to monitor fetal movements. There was no significant difference in the mean antepartum stillbirth rate per cluster in the intervention versus control group (2.90/1000 vs. 2.67/1000). The routine antenatal care guidelines of the UK National Institute for Health and Clinical Excellence (NICE) [31] that do not recommend fetal movement monitoring in uncomplicated pregnancies were largely dictated by the findings of this trial [32].

In addition, we identified four other intervention studies [33-36] and six observational studies [37-42]. A quasi-randomized trial by Neldam showed a statistically significant difference in antepartum stillbirth rates among women told to monitor fetal movements compared to those not being asked to monitor movements (0/1125 vs. 8/1125) [34]. The three before-after studies all showed a significant decline in stillbirth rates after formal introduction of fetal movement monitoring into clinical practice [33,35,36].

According to the observational study by De Muylder, high-risk women whose previously normal kick charts became abnormal had significantly higher antepartum stillbirth (194/1000 vs. 7/1000) and perinatal mortality (222/1000 vs. 27/1000) rates compared to women whose kick charts remained normal till delivery [37]. Other observational studies have mixed data regarding stillbirth outcome. Lema showed that poor fetal monitoring results had higher rates of stillbirths (5/27 vs. 1/83) [40], while a recent study by Sinha [42] found no deaths in the two groups of women with decreased and normal fetal movements (0/90 vs. 0/90) similar to the result of the study by Romero Gutierrez on perinatal mortality [41].

#### (2) Doppler velocimetry

Our literature search yielded 908 titles (Figure 2). Initially 25 studies were considered for inclusion in the review. Seven of these studies were excluded because the trial participants were described as ‘unselected population’ or of ‘low risk’ [43-49]. Two studies were excluded due to insufficient data [50,51]. Finally 16 studies were included in the review [52-67].

**Figure 3:**
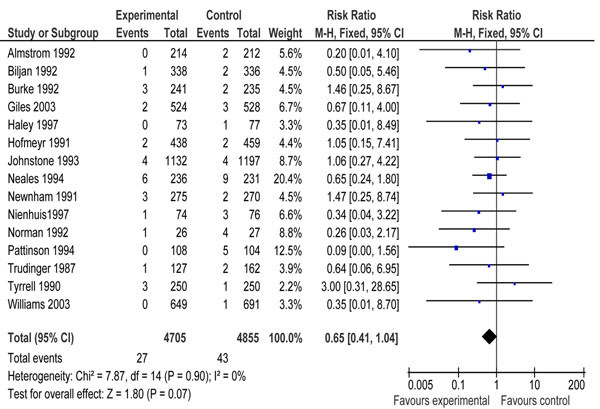
Forest plots for impact of Doppler ultrasound versus no ultrasound on stillbirths

**Figure 4:**
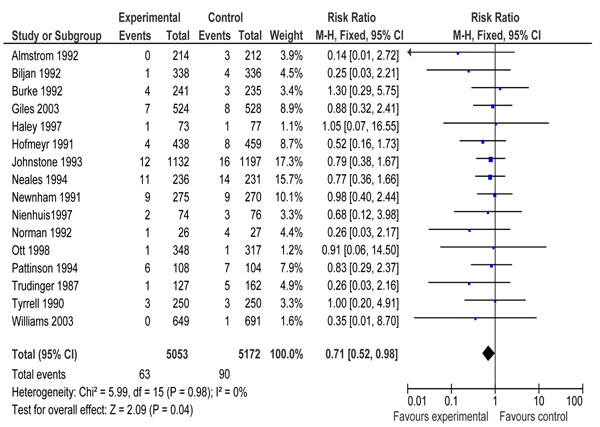
Forest plots for impact of Doppler ultrasound versus no ultrasound on perinatal mortality

Additional file 4 presents the characteristics of included studies of Doppler velocimetry. All the studies were conducted in high income countries except one that was conducted in South Africa [64]. Pooled results for impact on stillbirth showed a reduction of 35 % [RR 0.65, 95 % CI 0.41-1.04]; however the results did not reach the conventional limits of statistical significance (Figure 3). Pooled results from sixteen studies showed that Doppler velocimetry of umbilical and fetal arteries in ‘high risk’ pregnancies leads to a reduction of 29 % [RR 0.71, 95 % CI 0.52-0.98] in perinatal mortality compared to no Doppler velocimetry (Figure 4). There was no heterogeneity (I^2^ =0) in both the pooled estimates.

### Recommendations for LiST model

Table 1 gives an overall qualitative assessment of studies addressing fetal movement monitoring and Doppler velocimetry. Data were not pooled for fetal movement monitoring due to gross clinical heterogeneity in the intervention and control groups of the included studies. We have not recommended fetal movement monitoring for inclusion in the LiST model due to insufficient data in favor or against the use of intervention (GRADE quality very low).

For Doppler velocimetry, there was a significant reduction of 29% in perinatal mortality and non-significant reduction of 35% in stillbirths in high risk pregnancies. The results across studies were consistent in both estimates and there was no significant heterogeneity in the pooled data (I^2^ =0). The overall grade quality for reduction in perinatal mortality was that of ‘moderate’ level due to inadequate methods of sequence generation and allocation concealment in some of the included studies. Although the direction of effect (i.e. towards reduction) was similar for stillbirths, the overall grade quality of evidence for reduction in stillbirths was that of ‘moderate’ level. Keeping in mind the magnitude and direction of effect of these estimates, we recommend reduction in perinatal mortality [29 % (95 % CI 2% to 48%)] as a proxy for reduction in stillbirths with conversion of its overall quality grade from ‘moderate’ to ‘low’ level. This was to follow the theme of CHERG guidelines i.e. to select the most conservative estimate from the available data. The effect size for perinatal mortality (29%) was more conservative than that of stillbirth (35%). These recommendations can be interpreted as “Surveillance of high risk pregnancies with Doppler velocimetry of umbilical and fetal arteries with appropriate timely obstetric intervention leads to a reduction of 29 % (95 % CI 2% to 48%) in stillbirths”.

**Table 1:** Qualitative assessment of overall evidence for Doppler velocimetry and fetal movement monitoring according to CHERG rules

	Quality Assessment					Summary of findings		
				**Generalizability**		**Number of events**		Pooled Effect

**No. of studies**	**Design**	**Limitations**	**Consistency**	**Generalizability to Population of Interest**	**Generalizability to intervention of Interest**	**Intervention**	**control**	RR ( 95 % CI)

**Effect of surveillance of high risk pregnancies with Doppler velocimetry: Outcome perinatal mortality: Grade quality of evidence ‘Moderate’**								

16	RCT	Methods of sequence generation and allocation concealment were not adequate in most of the studies	No heterogeneity (I^2^=0%)	All the studies from developed countries except one which is from South Africa	Doppler velocimetry of umbilical and fetal arteries for surveillance of high risk pregnancy	63	90	0.71 (0.52-0.98)

**Effect of surveillance of high risk pregnancies with Doppler velocimetry: Outcome stillbirth: Grade quality of evidence ‘Low’**								

15	RCT	Methods of sequence generation and allocation concealment were not adequate in most of the studies	No heterogeneity (I^2^=0%)	All the studies from developed countries except one which is from South Africa	Doppler velocimetry of umbilical and fetal arteries for surveillance of high risk pregnancy	27	43	0.65 (0.41-1.04)

**Effect of fetal movement monitoring on stillbirths: Grade quality of evidence “very low”**								

14	RCT, quasi experimental and observational studies	Most of the evidence from observation studies. Of the four RCTs, only one compared fetal movement monitoring versus no fetal movement monitoring. This RCT showed no effect of fetal movement monitoring on stillbirths	Data not pooled due to gross clinical heterogeneity	Most of the studies from developed countries	No consensus on single counting method. Cardif method (Count to ten) was the most widely used method	Data not pooled		

## Discussion

### Detection and management of IUGR

Maternal BMI screening is one of the methods that have been suggested to predict growth of fetus and related occurrence of low birth weight, and other perinatal adverse outcomes [16,20,21,68-73]. A Cochrane review on effectiveness of measurement of symphysis fundal height for detecting IUGR was inconclusive due to lack of RCTs [19]. Observational studies however suggest that it is a cost effective and fairly accurate tool to detect or at least suspect abnormal fetal growth [15,23]. In case of clinical suspicion and/or existing risk factors, repeat ultrasound can assess fetal growth and a judgment can be made about optimal or suboptimal growth [15]. However, routine ultrasound for every woman irrespective of indication or risk factor does not help to reduce perinatal mortality [17,18].

Keeping in mind the existing literature reviewed elsewhere by us [15], we propose a model to detect and manage IUGR with an expected reduction in stillbirths. This model consists of three screening interventions i.e. maternal BMI, symphysis-fundal height measurement and targeted ultrasound coupled with management of cases identified. As there are currently no studies evaluating this combination, we consulted experts in the field to give us their opinion on the expected benefit of these combined interventions in reducing IUGR and stillbirths. Delphi consensus (medians) determined the effect to be 20% reduction in ante-partum stillbirth with an inter-quartile range of 10% to 37.5% and 20% reduction in intra-partum stillbirth with an inter-quartile range of 5% and 30% (Figure 1).

#### Surveillance of high risk pregnancies

Several surveillance methods have been proposed to detect and manage high risk pregnancy during the antenatal or intrapartum period [6]. These methods involve assessment of fetal well-being by taking into account measures such as fetal movement, fetal heart rate pattern, and/or growth; and feto-placental and/or uteroplacental circulatory dynamics [15].

There are no clearly identified criteria to distinguish between a ‘high’ or ‘low’ risk pregnancy; however, pregnancies in which the maternal and/or fetal condition pose a threat to life of the mother or fetus are considered as ‘high risk’[13]. Maternal conditions most commonly associated with adverse perinatal outcomes include conditions such as diabetes (chronic and gestational), hypertensive disorders (chronic hypertension and pre-eclampsia) and cardiac, renal, autoimmune and thrombophiliac disorders [74-76]. Fetal conditions associated with ‘high risk’ pregnancy include fetal growth restriction, and placental insufficiency [77-79].

Doppler velocimetry is considered as one of the most objective methods to assess fetal wellbeing in cases of intrauterine growth restriction (IUGR) [13,15]. It provides information on fetal and placental cardiovascular function on the basis of the blood flow dynamics measured in uterine, umbilical and fetal arteries [80]. A Cochrane review by Alfirevic et al. comprising of 16 studies and involving 10, 225 babies had shown that fetal and umbilical artery Doppler ultrasound in high risk pregnancies can decrease the perinatal mortality by 29 % (RR 0.71, 95 % CI 0.52-0.98), when obstetric services were in place to ensure safe and timely delivery of the baby when needed. Uterine artery Doppler waveform analysis on the other hand, may identify compromised fetuses at risk of stillbirth, especially in cases of placental underperfusion associated with preeclampsia and/or growth restriction; however published literature does not show its effectiveness of subsequent intervention to prevent stillbirths [15]. We had therefore focused on effectiveness of Doppler velocimetry of fetal and umbilical arteries for the fetal wellbeing in case of surveillance of IUGR.

According to CHERG rules, we recommended a reduction of 29 % (95 % CI 2% to 48%) in stillbirths for high risk pregnancies if these are identified, followed by Doppler velocimetry of fetal and umbilical arteries and managed with the appropriate intervention (e.g. early delivery). This estimate was the most conservative of the estimates for reduction in perinatal mortality and stillbirths. The results for reduction in stillbirths did not reach statistical significance. The overall grade quality for the pooled estimate for still births was ‘low’. This was because the quality of methods of sequence generation and allocation concealment was inadequate in some of the included studies. We therefore propose to take reduction in perinatal mortality as a proxy for reduction in stillbirths. Our results are in accordance with the previous meta-analysis done on this topic [13]. It is important to take into account that Doppler ultrasound is used as a diagnostic assessment method and the clinical outcomes depend on availability of and implementation of timely interventions such as early delivery e.g. via caesarean sections.

Fetal movement counting is a simple, inexpensive and the oldest way to monitor the condition of the baby during pregnancy and is considered to be an indirect measure of central nervous system integrity [42,81]. Fetal movements in the womb can be felt by the mothers from around 16 to 20 weeks of gestation [82]. A reduction in fetal movements is associated with decreased oxygenation, which may lead to fetal growth compromise or stillbirth [83]. A review based on twenty-four Western studies demonstrated that reduced fetal movements were associated with adverse pregnancy outcomes, both in high and low risk pregnancies [84]. Therefore, decreased fetal movements may be a sign of fetal compromise or impending fetal demise. Other causes of reduced fetal movements include decreased amniotic fluid, drugs, sedatives and sleep state in the fetus [85]. A Cochrane review by Mangesi and Hofmyer, comprising four randomized controlled trials and including 71,370 women, found no convincing evidence to recommend in favor or against routine fetal movement monitoring in unselected or high risk pregnancies [86].

In developing countries, where advanced facilities are not available, fetal movement monitoring may be feasible, but its use is currently not supported by scientific evidence. We have graded the current evidence as ‘very low’ which means that there is not sufficient evidence to include this intervention in the LiST model. We however consider it important to study this simple and oldest intervention in more detail to assess if it is useful to detect and follow high risk pregnancies especially in developing countries.

## Conclusions

In conclusion, Detection and management of IUGR using maternal BMI screening, symphysis-fundal height measurement and targeted ultrasound could be effective method of reducing IUGR related stillbirths. There are currently no studies available to assess the effect of these methods. Based on the opinion of experts in the field, this combination coupled with effective management could reduce IUGR related antepartum and intrapartum stillbirth by 20% each.

Doppler velocimetry of umbilical and fetal arteries for surveillance of identified high risk pregnancies leads to a reduction of 29% (95 % CI 2% to 48 %) in perinatal mortality. The direction of effect on the incidence of stillbirths was also similar but not statistically significant [RR 0.65, 95 % CI 0.41-1.04]. We recommend an estimate reduction of 29 % (95 % CI 2% to 48%) in stillbirths for inclusion in the Lives Saved Tool on the basis of rules developed by Child Health Epidemiology Reference Group. There is insufficient evidence to recommend in favor or against of fetal movement counting. More research is needed to study this method especially in developing countries.

### Key messages

Doppler Velocimetry of umbilical and fetal arteries in women with high risk pregnancies leads to a reduction of 29 % (95 % CI 2% to 48 %) in perinatal mortality.

Pooled results for impact of Doppler Velocimetry on stillbirth show a statistically non-significant reduction of 35 % [RR 0.65, 95 % CI 0.41-1.04].

According to Child Health Epidemiology Group, we have recommended reduction in perinatal mortality by [29 % (95 % CI 2% to 48 %)] as a proxy for reduction in stillbirths in high risk pregnancies. It is important to take into account that Doppler ultrasound is a screening test and cannot influence clinically important outcomes itself. The clinical outcomes depend on availability of appropriate facilities to manage the patient.

Detection and management of IUGR using maternal BMI screening, symphysis-fundal height measurement and targeted ultrasound followed by appropriate management can be an effective method of reducing IUGR related stillbirths. Based on the opinion of experts in the field, this combination could reduce antepartum and intrapartum stillbirth by 20%.

## Competing interests

The authors declare no conflict of interest.

## Authors’ contributions

Professor Zulfiqar A Bhutta developed the review parameters and secured support. Drs Aamer Imdad, Yawar Yakoob and Saad Siddiqui undertook the literature search, data extraction and analysis under the supervision of Professor Bhutta. Professor Zulfiqar A. Bhutta gave advice in all the aspects of the project and was the overall supervisor.

## Supplementary Material

Additional File 1 Word document containing the search terms used in the two searches. Click here for file

Additional File 2 An excel sheet that show the data extraction sheet for studies evaluating Doppler velocimetry and fetal movement monitoring, as well as the CHERG rules. Click here for file

Additional File 3 A word document that shows the characteristics of included studies table: Fetal movement monitoring Click here for file

Additional File 4 A word file that shows the characteristics of included studies table: Doppler velocimetry Click here for file
