# Supplementary material for: Screening and triage of intrauterine growth restriction (IUGR) in general population and high risk pregnancies: a systematic review with a focus on reduction of IUGR related stillbirths
Source: BMC Public Health. 2011 Apr 13;11(Suppl 3):S1. doi: 10.1186/1471-2458-11-S3-S1 (PMC3231882; doi:10.1186/1471-2458-11-S3-S1)
Supplement: Additional File 1 — Word document containing the search terms used in the two searches. [file 1471-2458-11-S3-S1-S1.docx]

**Additional File 1: Search Terms**

**Appendix 1**

("Fetal Movement"[Mesh] OR "Fetal Monitoring"[Mesh] OR "fet* movement" OR "kickcharts" OR "fetal counting" OR "cardiff chart" OR "count to ten") AND ("Stillbirth"[Mesh] OR "Fetal Death"[Mesh] OR "Fetal Mortality"[Mesh] OR "Embryo Loss"[Mesh] OR "Fetal Viability"[Mesh] OR "Perinatal Mortality"[Mesh] OR stillbirth* OR "Fetal Death" OR "fetal loss" OR "perinatal mortality" OR miscarriage* OR abortion* OR "bab* death" OR "death of bab*" OR "infant death*" OR "neonat* death" OR "death of fetus" OR "intrauterine death*")

**Appendix 2**

("Ultrasonography, Doppler, Duplex"[Mesh] OR "Ultrasonography, Doppler, Pulsed"[Mesh] OR "Ultrasonography, Doppler, Color"[Mesh] OR "Ultrasonography, Doppler"[Mesh] OR doppler) AND ("Pregnancy"[Mesh] OR "Mothers"[Mesh] OR maternal OR pregnan* OR mother*) AND ("Stillbirth"[Mesh] OR "Perinatal Mortality"[Mesh] OR "Fetal Death"[Mesh] OR "Fetal Mortality"[Mesh] OR "Embryo Loss"[Mesh] OR "Fetal Viability"[Mesh] OR stillbirth* OR "fetal death*" OR "Fetal loss" OR "perinatal mortality" OR miscarriage* OR abortion* OR "bab* death" OR "death* of bab*" OR "infant death" OR "death of fetus" OR "intrauterine death*")
