# Supplementary material for: Screening and triage of intrauterine growth restriction (IUGR) in general population and high risk pregnancies: a systematic review with a focus on reduction of IUGR related stillbirths
Source: BMC Public Health. 2011 Apr 13;11(Suppl 3):S1. doi: 10.1186/1471-2458-11-S3-S1 (PMC3231882; doi:10.1186/1471-2458-11-S3-S1)
Supplement: Additional File 3 — A word document that shows the characteristics of included studies table: Fetal movement monitoring [file 1471-2458-11-S3-S1-S3.docx]

**Additional File 3: Characteristics of included studies table: Fetal movement monitoring**

| Study ID | Country | Type of study | Community or hospital setting | Population of study (high risk or low risk) | Intervention method used | Comparison Group | Grade quality |
| --- | --- | --- | --- | --- | --- | --- | --- |
| Gomez et al. 2003 [44] | Peru | RCT | Women attending single antenatal clinic | High risk pregnant women with > 30 weeks gestation. | The intervention group was to use fetal movement counting method proposed by Latin American Centre for Perinatology and Human Development | The control group was to monitor fetal movements via count to ten charting method | Moderate |
| Freda et al. 1993 [43] | USA | RCT | Women attending an antenatal clinic | Women attending an antenatal clinic with uncomplicated singleton pregnancy between 28-32 weeks of gestation | The Sadovsky fetal movement counting method | Women were allocated to either the Cardiff ’count-to-ten’ method | Moderate |
| Thomson et al. 1990 [42] | Denmark | RCT | Women attending an antenatal clinic | Women without obstetric complications and medical diseases were recruited at 16 to 18 weeks of pregnancy. | Monitoring of fetal movements by Cardiff ’count-to-ten’ method | Hormonal analysis group estriol and human placental lactogen were measured by radio-immunoassay at 33 weeks, 36 weeks, 39 and 41 weeks | Moderate |
| Grant et al. 1989 [40] | England | RCT | Women attending an antenatal clinic | Women with pregnancies between 28 and 32 weeks | Treatment group were women counting their fetal movements formally every day using a ’count-to-ten’ chart (Cardiff ) | Women in the control group were not told to monitor fetal movements but were asked about fetal movements on each antenatal visits and were allowed to raise concerns | High |
| Moore et al. 1989 [47] | USA | Before After Design | Hospital | Unselected population attending an antenatal clinic | Formal fetal movement assessment | No Formal assessment of fetal movements | Low |
| Nedlam 1980 [48] | Denmark | Quasi experimental | Hospital | Women with gestation age > 32 weeks | Women who were told to monitor Fetal movements three times a week | Women who were not told to regularly monitor Fetal movements | Low |
| Westgate et al. [49] | New Zealand | Before After Design | Hospital | Pregnant Women | Stillbirths after introduction of Fetal movement monitoring | Stillbirths before introduction of Fetal movement monitoring | Low |
| Eggertsen et al. [52] | USA | Observational Study | Hospital | Women monitoring Fetal Movements (n = 394) | Women with decreased fetal movements | No Comparison Group | Very Low |
| Saastad et al. 2010 | Norway | Before after design | Hospital | Singleton women presenting with decreased fetal movement in the third trimester across 14 hospitals in Norway | A brochure was distributed to the women in the intervention group. “The brochure covered information on: expected normal fetal activity; differences in perception according to different fetal movements, maternal position , the inter- and intra-individual variation between fetuses , maternal weight, and smoking ; interpretation of variation of fetal activity; instructions on how to use the kick chart; and when to contact health professionals if experiencing DFM”. | Routine care for decreased fetal movements. | Low |
| De Muylder[51] | Zimbabwe | Observational | Hospital | Pregnant women aged 17-42 years | Pregnant women whose kick charts became abnormal | Pregnant women whose kick chart remained normal till delivery | Very low |
| Lema et al.[54] | Kenya | Observational | Hospital | Antenatal patients with gestation age >32 weeks | Pregnant women with poor Daily Fetal Monitoring Chart Results | Pregnant women with good Daily Fetal Monitoring Chart Results | Very low |
| Sinha et al.[33] | UK | Observational | Hospital | Antenatal patients with gestation age >24 weeks | Pregnant women with complaints of Decreased fetal movements. | Pregnant women with normal fetal movements | Very low |
| Romero Gutiérrez et al.[55] | Mexico | Observational | Hospital | Pregnant women with 32-41 weeks gestation period and no other risk factors | Women with decreased fetal movements | Pregnant women with normal fetal movements | Very low |
| Eggertsen et al.[52] | Not available | Observational | Hospital | Women monitoring Fetal Movements (n = 394) | Women with decreased fetal movements | Women with no complains of decrease fetal movements | Very low |
| Valentin et al.[53] | Sweden | Observational | Antenatal Services | Pregnant women who were instructed to count Fetal movements throughout third trimester of pregnancy. | Women that consulted for decreased Fetal movements | Women that did not consult for decreased fetal movements. | Very low |
