# Supplementary material for: Screening and triage of intrauterine growth restriction (IUGR) in general population and high risk pregnancies: a systematic review with a focus on reduction of IUGR related stillbirths
Source: BMC Public Health. 2011 Apr 13;11(Suppl 3):S1. doi: 10.1186/1471-2458-11-S3-S1 (PMC3231882; doi:10.1186/1471-2458-11-S3-S1)
Supplement: Additional File 4 — A word file that shows the characteristics of included studies table: Doppler velocimetry [file 1471-2458-11-S3-S1-S4.docx]

**Additional File 4: Characteristics of included studies table: Doppler velocimetry**

| Study ID | Country | Type of study | Community or hospital setting | Population of study (high risk or low risk) | Intervention method used | Comparison Group | Quality Grade |
| --- | --- | --- | --- | --- | --- | --- | --- |
| Almstrom et al. 1992 [65] | Sweden | RCT | Hospital | Women with fetuses found to be small on ultrasound examination at 31 completed weeks of pregnancy or later | Antenatal surveillance with Doppler velocimetry. | Antenatal surveillance with cardiotopography | Low |
| Biljan et al. 1992 [66] | UK | RCT | Hospital | Women with high-risk singleton pregnancies. | Revealed Doppler of umbilical artery | concealed Doppler of umbilical artery | Low |
| Burke et al. 1992 [67] | Ireland | RCT | Hospital | Pregnant women With ‘high risk’ pregnancies. Risk assessment was based on suspected IUGR, hypertensive disorders, previous  baby < 2.5 kg, antepartum hemorrhage, previous perinatal death, diminished fetal movements, post maturity, diabetes and others | Umbilical artery Doppler and | Fetal biometry and biophysical profile (BPP) score only | Low |
| Giles et al. 2003[68] | Australia, New Zealand and Southeast Asia | RCT | Hospital | Pregnant women with twin pregnancies at 25 weeks gestation | Standard ultrasound biometric assessment plus Doppler ultrasound umbilical artery flow velocity waveform analysis | Standard ultrasound biometric assessment | Moderate |
| Hofmeyr et al. 1991 [70] | UK | RCT | Hospital | Women with ‘high risk’ pregnancy | Doppler ultrasound of umbilical artery. | "Fetal Heart Rate" group. 39% of the controls underwent Doppler exam | High |
| Haley et al. 1997 [69] | UK | RCT | Hospital | Women with abdominal  circumference < 2 SD shown on ultrasound with reference to mean for the gestational age fetal heart rate on charts recommended by British Medical Ultrasound Society. All women were > 26 weeks gestation. | Umbilical artery Doppler and CTG | CTG only | High |
| Johnstone et al. 1993 [71] | UK | RCT | Hospital | Pregnancies defined as being at risk by referral for Doppler or fetal monitoring | Umbilical artery Doppler and other tests like CTG and BPP | No Doppler but usual monitoring by CTG and BPP | Moderate |
| Neales et al. 1994 [72] | UK | RCT | Hospital | Women at 24 wks or more with a singleton pregnancy with a fetus with an abdominal circumference <5th percentile on ultrasound measurement. | Doppler exam of the umbilical artery was performed and the results were revealed to the clinician | Doppler exam of the umbilical artery was performed however the results were concealed from the clinician | Moderate |
| Newnham et al. 1991 [73] | Australia | RCT | Hospital | Women with pregnancy abnormalities referred to an ultrasound department for fetal examination during the third trimester | Continuous wave Doppler studies of umbilical and uteroplacental arterial circulations. Results were revealed to patients and clinicians | Routine antenatal care | High |
| Nienhuis et al. 1997[74] | Netherlands | RCT | Hospital | Women with singleton pregnancies with clinical suspicion of fetal growth restriction | Doppler of the umbilical artery | No Doppler (39% of controls underwent Doppler examination) | High |
| Norman et al. 1992 [75] |  | RCT | Hospital | Women with high-risk pregnancies and at least 24 weeks pregnant. The risk assessment was based on recurrent pregnancy loss (2 or more mid trimester) or early third trimester losses which resulted in IUFD, stillbirth or neonatal death. | Doppler of the umbilical artery revealed | Doppler of the umbilical artery concealed | Low |
| Ott et al. 1998 [76] | USA | RCT | Hospital | Women with high risk pregnancies. The risk assessment was based on uteroplacental insufficiency, postdates; maternal diabetes; premature rupture of membrane, fluid abnormalities. | Fetal and umbilical Doppler + modified BPP. | No Doppler but modified BPP. | Moderate |
| Pattinson et al. 1994 [77] | South Africa | RCT | Hospital | Women with pregnancies 28 or more weeks gestation with hypertensive diseases and/or who were suspected of having small for gestational age fetuses, and were referred for Doppler examinations | Doppler velocimetry of the umbilical artery was performed using continuous wave Doppler and the results were revealed to the clinician | Doppler velocimetry of the umbilical artery was performed using continuous wave Doppler however the Doppler velocimetry results were withheld from the clinician | Low |
| Trudinger et al. 1987[78] | Australia | RCT | Hospital | Patients with singleton pregnancy admitted to antenatal ward (high fetal risk pregnancies) with gestation past 28 weeks (mean gestational age 34 weeks) | Doppler ultrasound examination of umbilical artery flow velocity waveforms | Routine antenatal care | Moderate |
| Tyrrell et al. 1990 [79] | UK | RCT | Hospital | Women with ‘high risk’ pregnancies. Risk assessment was based on suspicion of IUGR, Previous SGA, Hypertension and antepartum hemorrhage. Women with twin pregnancies and suffering from diabetes were excluded. | Doppler plus modified biophysical profile exam | No Doppler (4.8% of controls had Doppler for clinical indications.) | Moderate |
| Williams et al. 2003 [80] | USA | RCT | Hospital | Women with singleton high-risk pregnancies. Risk assessment was based on IUGR, hypertension, diabetes  , prolonged pregnancy, decreased fetal movements. | Umbilical artery Doppler | Electronic FHR with NST | Moderate |
